# Reliability and validity of OpenPose for measuring hip-knee-ankle angle in patients with knee osteoarthritis

**DOI:** 10.1038/s41598-023-30352-1

**Published:** 2023-02-25

**Authors:** Yoshitomo Saiki, Tamon Kabata, Tomohiro Ojima, Yoshitomo Kajino, Daisuke Inoue, Takaaki Ohmori, Junya Yoshitani, Takuro Ueno, Yuki Yamamuro, Atsushi Taninaka, Tomoyuki Kataoka, Naoyuki Kubo, Seigaku Hayashi, Hiroyuki Tsuchiya

**Affiliations:** 1 Department of Orthopaedic Surgery, Graduate School of Medical Sciences, Kanazawa University, 13-1 Takaramachi, Kanazawa City, Ishikawa 920-8641 Japan; 2 Department of Rehabilitation Physical Therapy, Faculty of Health Science, Fukui Health Science University, 55-13-1 Egami, Fukui City, Fukui 910-3190 Japan; 3 Department of Orthopaedic Surgery, Fukui General Hospital, 58-16-1 Egami, Fukui City, Fukui 910-8561 Japan; 4 Department of Rehabilitation Physical Therapy, Fukui General Hospital, 58-16-1 Egami, Fukui City, Fukui 910-8561 Japan

**Keywords:** Musculoskeletal abnormalities, Osteoarthritis

## Abstract

We aimed to assess the reliability and validity of OpenPose, a posture estimation algorithm, for measuring hip-knee-ankle (HKA) angle in patients with knee osteoarthritis, by comparing it with radiography. In this prospective study, we analysed 60 knees (30 patients) with knee osteoarthritis. We measured HKA angle using OpenPose and radiography before or after total knee arthroplasty and assessed the test–retest reliability of each method with intraclass correlation coefficient (1, 1). We evaluated the ability to estimate the radiographic measurement values from the OpenPose values using linear regression analysis and used intraclass correlation coefficients (2, 1) and Bland–Altman analyses to evaluate the agreement and error between OpenPose and radiographic measurements. OpenPose had excellent test–retest reliability (intraclass correlation coefficient (1, 1) = 1.000) and excellent agreement with radiography (intraclass correlation coefficient (2, 1) = 0.915), with regression analysis indicating a large correlation (R^2^ = 0.865). OpenPose also had a 1.1° fixed error and no systematic error when compared with radiography. This is the first study to validate the use of OpenPose for the estimation of HKA angle in patients with knee osteoarthritis. OpenPose is a reliable and valid tool for measuring HKA angle in patients with knee osteoarthritis. OpenPose, which enables non-invasive and simple measurements, may be a useful tool to assess changes in HKA angle and monitor the progression and post-operative course of knee osteoarthritis. Furthermore, this validated tool can be used not only in clinics and hospitals, but also at home and in training gyms; thus, its use could potentially be expanded to include self-assessment/monitoring.

## Introduction

Hip-knee-ankle (HKA) angle is widely used as a measurement of knee alignment in knee osteoarthritis (OA). The HKA angle is defined as the angle between the mechanical axes of the femur and tibia in the coronal plane^1^. Based on this angle, lower extremity alignment is classified as varus, neutral or valgus^2–4^. Many reports have shown that varus or valgus alignment increases the risk of incidence and progression of knee OA^5–9^. Moreover, surgical treatment of knee OA, such as osteotomy and arthroplasty, is important to reconstruct optimal alignment and correct load distribution in the medial and lateral compartments. In total knee arthroplasty (TKA), excessive postoperative varus alignment has been reported to have a negative impact on patient satisfaction and implant longevity^10–14^. Therefore, the measurement of the HKA angle is important throughout the entire course of knee OA.

The HKA angle is measured from a full-length lower-limb radiograph in a standing position. Full-length radiographs involve the risk of radiation exposure, additional technician training, increased time for image acquisition, and increased overall cost. Therefore, methods to estimate the HKA angle without full-length radiographs have been discussed. One such method is to estimate the HKA angle from a standard anteroposterior (AP) knee radiograph^15,16^. However, the difference between the anatomical and mechanical axes of the femur and tibia varies across patients, and this difference may reduce the accuracy of the estimation. In addition, since radiation exposure is unavoidable, routine measurement of the HKA angle is difficult. The development of a simple method for measuring the HKA angle without radiation exposure will allow quantitative routine evaluation of the progression of knee OA and its postoperative course.

Conventional motion analysis has mainly used inertial motion capture systems and optical marker motion capture systems. Marker motion capture requires optical reflective markers and has high measurement accuracy, but also requires a lot of space, time and cost. Recently, markerless motion capture methods, such as methods to create three-dimensional (3D) images from multiple camera images^17^, methods using depth sensors^18,19^ and methods using deep learning^20,21^, have been validated for accuracy and clinical application. OpenPose, an algorithm for estimating two-dimensional (2D) human poses in images using deep learning, is one such method^22,23^. OpenPose can estimate body feature points from photographs and videos using a standard digital camera without special measurement equipment or environment. This open-source software is available for free non-commercial use and is increasingly being applied in the field of motion analysis because of its ease of implementation^20,24,25^. These reports on healthy participants have suggested that OpenPose could be a useful tool for measuring knee joint range of motion in the sagittal plane. However, the measurement accuracy of the HKA angle in the coronal plane using OpenPose has not been validated. Furthermore, it is important in terms of clinical application to validate the accuracy of OpenPose for measuring HKA angle in deformed lower limbs, such as osteoarthritic knees, not just healthy knees. Verification of its reliability and validity may lead to the establishment of a new, simple, and inexpensive measurement tool in clinical practice and at home. This verification also has the potential to make it easier than ever to investigate changes in knee alignment over time.

Therefore, this prospective study aimed to clarify the reliability and validity of HKA angle measurements using OpenPose pre- or post-operative TKA compared with radiography. We hypothesised that OpenPose would be a highly reliable and valid HKA angle measurement tool.

## Methods

### Study design and patients

In this prospective study, we included patients who underwent TKA for knee OA between April 2021 and February 2022. The sole exclusion criterion was inability to maintain a standing position because this might interfere with accurate measurements. However, none of the cases met this exclusion criterion. A total of 60 knees (30 patients) were assessed with pre- or post-operative radiographs. Out of the 60 knees with OA, 35 were TKA knees and 25 were non-TKA knees. Patients were assessed either pre- or post- operatively, not both. All procedures were performed using a cruciate-retaining Persona knee system (Zimmer Biomet Inc., Warsaw, IN) by a single surgeon at a single institution. This study was approved by Nittaduka Medical and Welfare Center Ethics Review Committee and performed according to the principles of the Declaration of Helsinki. All patients provided written informed consent.

We used GPower 3.1.9.2 to determine the appropriate sample size. In the linear regression analysis, a sample size of 55 participants was required to obtain an effect size (f^2^) greater than 0.15 with 0.80 power and 0.05 error level on two-tailed test. Therefore, this study enrolled 60 knees. Participants were asked to wear plain trousers of appropriate size to prevent misinterpretation of OpenPose measurements.

### Data collection and analysis

We collected two types of imaging data for HKA angle estimation, first with radiography then with OpenPose, pre- or post-operatively. We obtained AP full-length radiographs with the patient in a standing position with the knee extended and the patella facing forward. The X-ray beam was perpendicular to the plane of the leg and at the level of the knee joint. Next, we obtained coronal images of the lower extremity using a digital camera immediately after the radiography. The patient’s posture and imaging direction were the same as for the radiography; the images were taken perpendicular to the plane of the leg, at the level of the knee joint, so that the entire body was captured on screen.

**Figure 1:**
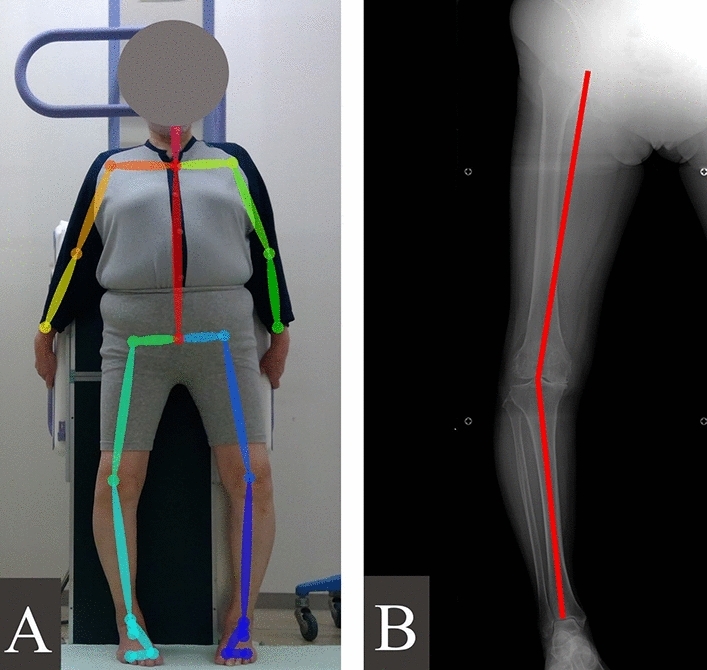
Images for hip-knee-ankle angle measurement. (**A**) OpenPose image. (**B**) Radiographic image.

The two types of images were then analysed. For radiography, the HKA angles were measured twice from the same radiographs with a 1-week interval between measurements. The average value of the two measurements was calculated. The HKA angle was defined as the angle between the femoral and tibial mechanical axis (Fig. 1A)^1^. The femoral mechanical axis was defined as a line connecting the centre of the femoral head and knee joint. The tibial mechanical axis was defined as a line connecting the centre of the knee and ankle joint. The HKA angles were expressed as degrees deviation from 180° and characterised as being varus (≤ − 3°), neutral (0 ± 3°), or valgus (≥ 3°)^3,4^. This measurement was conducted by two separate raters. To prevent information bias, the second rater was blinded to the OpenPose data. The data of the second rater were used as the representative values. The inter-rater reliability between the two raters was evaluated and found to be excellent (intraclass correlation coefficient [ICC] = 0.994); thus, the radiographic measurement by the second rater was deemed to be sufficiently reliable. For OpenPose, the feature points of each joint were estimated from the relevant images (Fig. 1B). The HKA angles were calculated from the estimated coordinates of the hip, knee, and ankle joints. These analyses were performed twice from the same image automatically by Python code, and the average values were compared with those of the radiographic measurements.

### Statistical analysis

The test–retest reliability was evaluated using ICCs (1, 1). ICC values of < 0.5, 0.5–0.75, 0.75–0.9, and > 0.9 indicated poor, moderate, good, and excellent agreement, respectively^26^. The OpenPose measurement was compared with radiographic measurement to assess its validity. Linear regression analysis was used to evaluate the possibility of estimating the radiographic value from the OpenPose value. R^2^ values of 0.01–0.09, 0.09–0.25, and > 0.25 indicated small, medium, and large correlations, respectively^20^. ICCs (2, 1) and Bland–Altman analysis were used to evaluate the agreement between the OpenPose and radiographic values^27,28^. The mean of the difference between the radiographic and OpenPose measurements is a fixed error, and the mean of the absolute difference between the radiographic and OpenPose measurements is an absolute error. All statistical analyses were performed using R software version 4.1.0 with a significance level of *p* < 0.05^29^.

## Results

We analysed 60 knees (22 men and 38 women; mean age: 73.8 ± 6.0 years; mean height: 156.8 ± 8.0 cm; mean mass: 64.7 ± 8.8 kg; body mass index: 26.3 ± 3.0 kg/m^2^; TKA knees: 35, OA knees: 25; varus knees: 18, neutral knees: 39, valgus knees: 3).

The mean (standard deviation [SD]) HKA angles were − 1.59° (5.67) and − 2.67° (5.90) for OpenPose and radiography, respectively. The mean (SD) of the difference between OpenPose and radiography is − 1.077° (2.171). The mean (SD) of the absolute difference between OpenPose and radiography is 1.799° (1.613). The test–retest reliability values (ICCs) of these two measurements are listed in Table 1. Both measurements showed excellent test–retest reliability.

**Table 1:** Test–retest reliability for OpenPose and radiography.

Measurement	Hip-knee-ankle angle (°)	ICCs (1, 1)	95% CI for ICCs
OpenPose	− 1.59 ± 5.67	1.000	1.000–1.000
Radiography	− 2.67 ± 5.90	0.996	0.994–0.998

Values are presented as mean ± standard deviation.

*ICCs* intraclass correlation coefficients, *CI* confidence interval.

**Figure 2:**
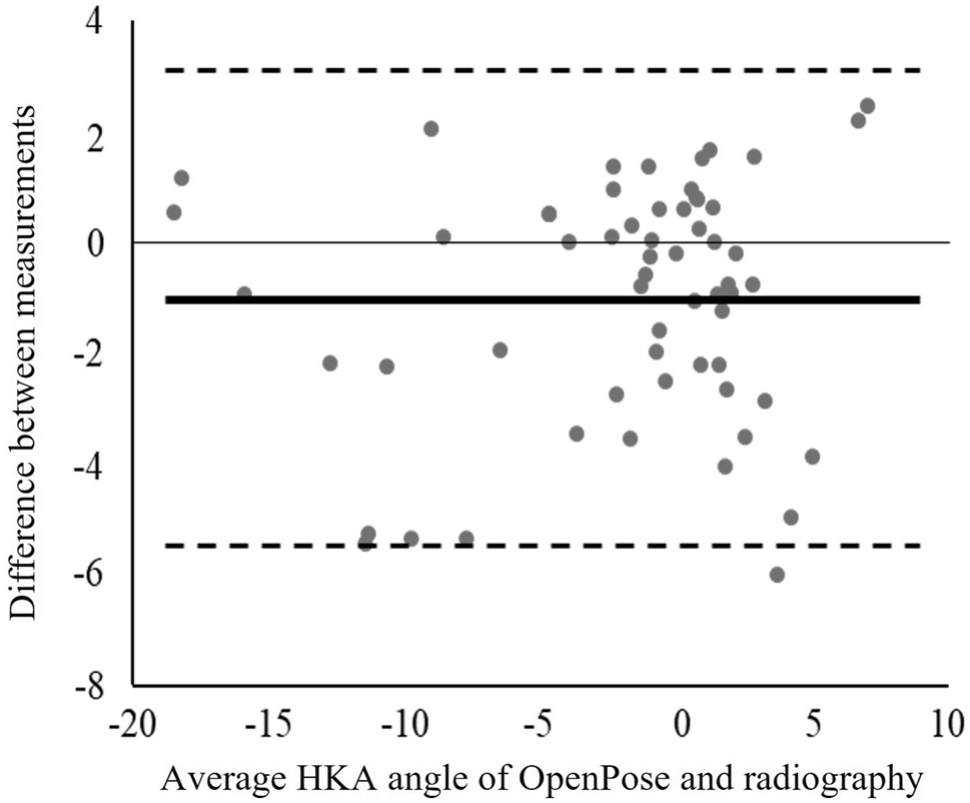
Bland–Altman plot for assessing measurement error. Mean hip-knee-ankle angle measurements for OpenPose and radiography are plotted on the x-axis and the difference between measurements on the y-axis.

The estimation possibility and agreement between the measurements are shown in Table 2. The regression analysis indicated a large correlation. The ICC (2, 1) between OpenPose and radiography indicated excellent agreement (0.915). Results of the Bland–Altman analysis and the Bland–Altman plot are shown in Table 3 and Fig. 2, respectively. A significant fixed error (mean difference [MD]) of − 1.077° was observed between OpenPose and radiography. No significant proportional error was observed between OpenPose and radiography. The random error (SD for MD) of OpenPose against radiography was 2.171.

**Table 2:** Regression models and intraclass correlation coefficients (2,1) between OpenPose and radiography.

Measurement	Coefficients B	95% CI for B	R^2^	ICCs (2, 1)	95% CI for ICCs
OpenPose × radiography	0.968	0.867–1.068	0.865	0.915	0.824–0.955

*CI* confidence interval, *R*^*2*^ coefficient of determination, *ICCs* intraclass correlation coefficients.

**Table 3:** Bland–Altman analysis between OpenPose and radiography.

Measurement	AD	MD	LOA	Fixed error		Proportional error	
			(lower–upper)	95% CI for MD	Coefficient B	95% CI for B	*p* value
OpenPose × radiography	1.799 ± 1.613	− 1.077 ± 2.171	8.511 (− 5.332–3.179)	− 1.638 to − 0.516*	0.041	− 0.059 to 0.141	0.418

Values are presented as mean ± standard deviation.

*AD* mean of absolute difference, *MD* mean of difference, *LOA* 95% limits of agreement, *CI* confidence intervals.

*Indicates that there was a significant fixed error.

## Discussion

This study investigated the clinical efficacy, reliability and validity of HKA angle measurement using OpenPose. The main findings of this study were that OpenPose had excellent test–retest reliability (ICC (1, 1) = 1.000) and excellent agreement with radiography (ICC (2, 1) = 0.915) for HKA angle measurement. The random error of OpenPose against radiography was small at 2.171. This study was the first to apply OpenPose to HKA angle measurement.

OpenPose uses models trained on a large number of images to predict a confidence map for each joint at each pixel in a 2D image^22^. OpenPose takes the maximum value of the confidence map to distinguish the accuracy of peaks and considers the pixel with the maximum value to be the joint position. This mechanical estimation of joint positions may allow for strongly reproducible measurements from the same image.

OpenPose measurement had large correlation and excellent agreement with radiography, thus confirming its validity. OpenPose had a fixed error of 1.077° underestimation compared to the gold standard full-length radiography, an absolute error of 1.799°, and a random error of 2.171°. Previous studies comparing the measurement of HKA angle using full-length radiographs vs standardized AP knee radiographs reported a fixed error of 1.8–4.4°, an absolute error of 2.5°, and a random error of 3.6°^15,16,30,31^. The misalignment of the mechanical and anatomical axes of the femur and tibia are thought to have produced these errors between standard AP and full-length radiographic measurements. Additionally, Vanwanseele et al.^32^ reported that HKA angle measurement by the marker-based 3D motion analysis system had a fixed error of 3.6° underestimation when compared with full-length radiographic measurement and a random error of 2.4°. This fixed error may be due to the method used to estimate the hip centre. The method used by Vanwanseele tends to estimate the hip centre externally, which could have resulted in a larger fixation error in the valgus direction^33^. Furthermore, the random error of the marker-based 3D motion analysis system was comparable to that of OpenPose in the present study. Therefore, in terms of validity and reliability, OpenPose has the potential to provide just as good if not better HKA angle measurements than other systems under controlled measurement tasks and conditions; OpenPose is close to the calculated values of the gold standard radiographic measurements for HKA angle measurements. OpenPose also underestimated HKA angle by 1.077° in the varus direction, which may be because it estimates joint position based on differences in the contrast of pixels around the joint. The hip joint, which has more soft tissue, may have larger estimation errors than the knee and ankle joints. However, the effect of underestimation can be reduced by offsetting − 1.077° from the output value. The mechanical axis after TKA is ideally within 0° ± 3° in terms of loosening^34^. Although an error of − 1.077° is not large, it has a SD of 2.171°. Therefore, without the offset of − 1.077° from the output value, more cases are likely to be incorrectly determined as having malalignment than with the offset. We thus encourage users to offset the output to improve the validity of OpenPose.

The accuracy of OpenPose estimation in this study was higher than in previous reports^20,24,35^. Ota et al. reported an ICC (2, 1) of 0.8 for maximum knee flexion angle in squatting and 0.62–0.67 for maximum knee flexion angle in walking on a treadmill with regard to the measurement validity of OpenPose compared with a 3D motion analysis system^20,24^. Yamamoto et al.^35^ similarly reported an ICC (2, 1) of 0.54–0.67 for maximum knee flexion angle during walking on a treadmill. Squatting differs from walking in that the positional relationship between the subject and the camera is less likely to change. The plane formed by the three joint points must be aligned with the plane to be measured to accurately measure joint angles with a single camera. Therefore, 2D images must be taken from a direction perpendicular to the plane formed by the three joint points to measure the joint angles accurately with a single camera. The image for OpenPose was taken perpendicular to the plane of the leg at the height of the knee joint, as in radiography. As a result, the OpenPose measurement may have had a high estimation accuracy. Therefore, if the task conditions (i.e., camera and subject positioning) are easy to control, OpenPose has the ability to produce highly accurate measurements, which increases its potential for clinical and research applications.

OpenPose measurements do not provide information regarding whether a lower extremity malalignment is due to intra- or extra-articular deformities; therefore, its use is not recommended when the aim is to evaluate intra-articular or extra-articular deformities. However, OpenPose allows for frequent measurements without radiation exposure and can play a valuable role in assessing the progression of knee OA and its post-operative course. In the future, OpenPose has the potential to be a cost-effective portable measurement tool suitable for the frequent evaluation of lower extremity alignment in clinical and home settings.

This study has several limitations. First, the sample size of valgus knee deformities was small. However, OpenPose estimates the knee joint position based on differences in pixel contrast around the knee joint. Therefore, whether the knee joint is positioned medially or laterally relative to the hip or ankle joint may not affect the estimation accuracy of varus or valgus deformities. In the future, additional validation should be carried for valgus deformities. Second, it was impossible to perform the radiographic and OpenPose imaging simultaneously. Nonetheless, we instructed patients not to move until the two measurements were completed, and the two measurements were performed consecutively. Furthermore, we ensured that the lower limbs did not move until these measurements were completed. Third, the two measurements were not performed in a randomised order. However, HKA angle measurements from the radiographic images were estimated by a second rater who was blinded to the OpenPose measurements. OpenPose measurement was also performed mechanically. Therefore, we believe that information bias was unlikely to have been introduced by the order of measurement. Fourth, the accuracy of OpenPose estimation may be affected by the background. To validation the impact that different backgrounds may have had on the results of the OpenPose measurement, we measured by transposing different backgrounds onto the images of patients. We found that there was good agreement in the results and minimal differences in the errors. Therefore, it is unlikely that the fact that the background of this experiment is a radiology room will diminish the clinical applicability of OpenPose.

In conclusion, this study supported our hypothesis that OpenPose is a reliable and valid tool for measuring HKA angles in patients with knee OA. OpenPose, which enables non-invasive and simple measurements, may be a useful tool to assess changes in HKA angle and monitor the progression and post-operative course of knee OA. Furthermore, this validated tool can be used not only in clinics and hospitals, but also at home and in training gyms, and thus has the potential for widespread application.

## Data Availability

The datasets used and/or analyzed during the current study are available from the corresponding author on reasonable request.
